# Policy and practice recommendations for residential addiction treatment: a qualitative investigation on increasing equity in treatment for Black patients

**DOI:** 10.1186/s13722-026-00677-z

**Published:** 2026-07-28

**Authors:** Corinne A. Beaugard, Natrina L. Johnson, Daneiris Heredia-Perez, Elizabeth Addison, Sheila E. Chapman, Avik Chatterjee, Christina S. Lee, Phillip Reason, Tayla Weeden, Tracey Weeden, Miriam Komaromy

**Affiliations:** 1 Grayken Center for Addiction, Boston Medical Center, 801 Massachusetts Ave Boston, Boston, MA 02119 USA; 2 Section of General Internal Medicine, Department of Medicine, Boston Medical Center, 801 Massachusetts Ave Boston, Boston, MA 02119 USA; 3 Division of Health and Society, Department of Medicine, UCSF Benioff Homelessness and Housing Initiative, University of California San Francisco, 2540 23rd Street, San Francisco, CA 94110 USA; 4 Boston University Chobanian and Avedisian School of Medicine, 72 E Concord St, Boston, MA 02118 USA; 5 Boston Healthcare for the Homeless Program, 780 Albany Street, Boston, MA 02118 USA; 6 Boston University School of Social Work, 264 Bay State Rd, Boston, MA 02215 USA; 7 Brockton Behavioral Health Center, 34 N Pearl St, Brockton, MA 02301 USA

**Keywords:** Addiction, Residential treatment, Racial equity, Culturally tailored care

## Abstract

**Background:**

Addiction treatment in the US is less effective for Black patients compared to their White peers. Barriers to appealing, effective, and equitable treatment exist both outside and within and treatment settings. Residential treatment is an important part of the continuum of care and is associated with improvement across substance use and life domains. However, this setting can be particularly uncomfortable for Black patients because it requires them to live in an environment that may not adequately support them.

**Methods:**

Building on a multi-year community-engaged project to improve addiction treatment for Black patients, we explored how to improve residential treatment for this population. We conducted interviews and focus groups to understand the elements of residential treatment that could be modified and to identify specific recommendations. First, we interviewed seven experts who were patients and/or treatment providers in residential treatment programs focused on Black patients. Next, we conducted five focus groups with leadership, staff, and Black patients in a short-term residential treatment facility to generate recommendations for practice at this site.

**Results:**

We organized policy recommendations into five domains: (1) Enhance representation of Black people and people with lived experience in treatment settings; (2) Conduct staff training to effectively support Black patients in treatment; (3) Create accountability across organizational structures; (4) Increase awareness and responsiveness to trauma; and (5) Create a welcoming treatment environment, utilizing strength-based behavioral reinforcement and culturally tailored programming.

**Discussion:**

Our recommendations span multiple aspects of treatment, including hiring protocols, facilities, and clinical approaches. Our work adds to the literature by providing a set of policy recommendations that address the unique features of residential treatment. Manifestations of anti-Black racism in the US within and outside of the medical system make these changes particularly salient for Black patients. Pilot studies are needed to evaluate recommendations’ effectiveness for key treatment metrics, including treatment continuity, substance use and related consequences, patient-provider trust, and discrimination.

**Supplementary Information:**

The online version contains supplementary material available at 10.1186/s13722-026-00677-z.

## Introduction

Structural racism permeates all facets of the United States (US) healthcare system [1–3], including addiction treatment [4, 5]. Efforts to reduce racism in addiction treatment are essential because Black individuals face a higher burden of substance-related morbidity and mortality and access treatment at lower rates compared to their White peers [6–9]. Nationally, Black people are less likely to receive medication for opioid use disorder [10–12] or alcohol use disorder and have less access to harm reduction services [13–16]. Additionally, Black people face relatively longer wait times to treatment [17, 18], receive fewer services in treatment [17], and are more likely to leave treatment early [12, 19, 20]. Many of these disparities are due to the long history of racism, racialized policies, and beliefs about substance use in the Black community [21]. 

Historically, problematic substance use was viewed as a moral failure or criminal act, which prompted religious and/or criminal-legal responses [22, 23]. As addiction science advanced over the twentieth century, the medical model came into fashion and was believed to de-stigmatize addiction by reducing the focus on personal responsibility [24, 25]. The medical – or brain disease – model of addiction located treatment within the biomedical sphere, downplaying the role of social factors in its development and treatment [26]. Though this model has since been challenged, it changed our approach to addiction treatment, shifting response from the moral and legal spheres to those of physical and mental health [24, 27]. This ideological transition benefitted White people who became viewed as patients needing treatment. Despite the broader reframing of treating addiction as a disease, the legacy of the War on Drugs and the criminalization of drug use continue to shape society’s punitive approach towards Black people who use drugs [21, 28]. 

Improving addiction treatment for Black people requires a thorough examination of the continuum of care and how it supports or does not support Black patients. To our knowledge, there is little research on Black patients’ experience in residential treatment. In other addiction settings (e.g., outpatient treatment) Black patients have described being treated with suspicion and apprehension when they express emotion [22] and stereotyped and reprimanded for perceived aggression [28]. Experiences of racism such as these deter Black people from engaging in treatment and consequently contribute to inequities in treatment outcomes [29, 30]. Additionally, treatment modalities that work for White patients may be less supportive for Black patients. For example, an ethnographic study found that White individuals in a residential therapeutic community (TC) were comfortable with the TC approach and the notion of adopting an “addict” identity [22]. Black individuals in the study were less likely to adopt this identity or participate in the TC requirements and thus did not receive the benefits conferred to their White peers.

Residential treatment is an effective part of the care continuum and provides treatment in a protected environment away from the stressors of community and home environments [31]. This setting is often preferred to outpatient treatment by people with more complex social needs and higher perceived risk of return to use [32, 33]. Modifications to this setting, however, are needed to facilitate engagement from populations who could benefit from this level of care. This study is part of a larger study (the parent project) that consulted with community partners and subject matter experts on how to improve addiction treatment for Black patients [34]. During the earlier phases of our work, we learned residential treatment was especially challenging for Black patients and deserved focused attention. Our primary aim in this study was to generate focused policy and practice recommendations to improve residential treatment for Black patients.

## Methods

### Short term residential treatment site

This study was conducted at Boston Medical Center, the largest safety net hospital in New England. The hospital operates a short-term residential addiction treatment center (< 30 days). This program serves a diverse patient population, and its program leadership and staff had substantial experience working with Black patients. We approached the Executive Director about conducting the study at this site. The Executive Director agreed, as she was invested in improving Black patients’ experience in the program. She participated in the early research stages, supported the grant writing, provided edits, and offered final approval.

### Protocol development

Team members [NLJ, DHP, MK] drafted the interview and focus group guides, which were subsequently reviewed by the full team. Our team included addiction medicine and primary care physicians, epidemiologists, a community engagement specialist, social workers, and public health professionals. Team members primarily identified as Black, and some had personal experiences with addiction and recovery.

We designed the expert interview protocol to gather information about treatment that had been tailored for Black patients. We asked these experts to provide examples of tailoring at their programs and their perspectives on how these efforts influenced Black patients' treatment experiences. We modified the protocol depending upon the individual’s experience (i.e., only professional OR both patient and professional). The focus group protocols for the leadership, staff, and Black patients were focused on this specific short-term residential facility. We asked each group to describe the policies and practices at the short-term residential facility that contributed to patient success, the role of patient race in treatment experience, and recommendations to improve the program for Black patients. Protocols provided in Appendix A.

### Data collection: interviews and focus groups (Feb-Apr 2024)

#### Expert interviews

We conducted expert interviews to understand what worked historically in treatment programs focused on Black patients. We recruited seven experts through our professional networks and snowball sampling. These individuals were the foremost experts on the history of addiction treatment for Black people in Massachusetts, and some had been affiliated with the two former residential facilities designed for Black patients. These settings remain influential in the history of addiction treatment in Boston, especially for Black individuals. Their roles included: (1) four current and/or former directors of addiction treatment centers; (2) a current director of a recovery center; (3) a treatment consultant and recovery coach; and (4) a recovery coach. All experts were Black and included three men and four women; some had both lived addiction and recovery experience, as well as professional expertise.

Three team members [CB, DHP, MK] conducted the expert interviews in pairs; one led the interview and the other took notes. The interviews lasted approximately one hour, were conducted virtually, and were recorded for professional transcription. We emailed the consent document in advance of the interview.

#### Focus groups

We conducted a focus group with leadership at the short-term residential program. The PI (MK) and project manager (DHP) jointly led this focus group. Leadership roles included the Executive Director (TrW), Director of Operations, and Recovery Specialist Supervisor. Leadership participants were female and two identified as Black and one as White. We held this one-hour focus group in person at the treatment facility, and recorded it for professional transcription. We emailed the consent document prior to the focus group.

The community engagement specialist (CM) and project manager (DHP) co-facilitated the two staff focus groups. Staff roles spanned the program positions and included Behavioral Health Counselors, a Recovery Nurse, a Recovery Specialist, a Recovery Case Manager, and a Discharge Planner. The ten staff participants included male and female individuals who identified as Black, White, and Hispanic. We held these one-hour focus groups in person at the treatment facility and virtually, and we recorded them for professional transcription. We emailed consent prior to the focus groups.

We conducted two patient focus groups and invited all Black patients at the short term residential treatment program to participate. The community engagement specialist (CM) and project manager (DHP) co-facilitated patient focus groups. Staff informed patients about the focus groups by announcing the opportunity during meetings and by hanging fliers in common spaces. The fliers and announcements included the study’s eligibility criteria: (1) identify as Black; (2) ≥ 18 years old; and (3) can consent in English. A total of 18 patients participated, including male and female individuals. We held these 90-minute focus groups in person at the treatment facility, and we recorded them for professional transcription. Prior to beginning focus groups, we reviewed consent documents with patient participants and received verbal consent.

Member checking: After the series of interviews and focus groups, we drafted an initial set of recommendations. We reconvened with stakeholders to discuss our findings and elicit feedback to assure our interpretation accurately reflected participants’ perspectives [35]. We held three member checking groups, one with leadership at the short-term residential facility, one with staff at the short-term residential treatment, and one with Black patients who were in treatment at the time of the member checking group. We discussed the recommendations with each group and sought their feedback. After this round of revisions, we shared an updated version with the community advising board from our parent project [36]. We made final refinements after meeting with our community advising board. Human Ethics and Consent to Participate declarations: The Boston Medical Center and Boston University Medical Campus Institutional Review Board determined this study was exempt.

#### Analysis

A team member trained in qualitative methods [CAB] led and supervised the analysis. Our coding process was simultaneously inductive and deductive. We started with a deductive code book based on the Major Topic Areas (MTA) framework developed in the parent project. The framework was developed during a series of large group meetings with academic researchers, clinicians, frontline staff, and people with lived experience. We conducted rigorous qualitative analysis of meeting transcripts to identify the most salient recommendations to improve addiction treatment for Black patients across the continuum of care. The process of MTA development is described in full in our parent project paper [34]. MTAs included (1) hire Black staff; (2) offer staff training and development to work with Black patients; (3) require institutional accountability; (4) eliminate punitive policies; (5) treat trauma; (6) address structural barriers; (7) provide culturally tailored care. Each MTA code included examples from the parent project.

The coding team included four team members who met weekly to evaluate the codebook, discuss the coding process, and refine codes. When the MTA codebook did not account for emergent themes, we developed new codes through a process of open coding. We discussed these data during team meetings and incorporated new codes into our codebook. Two people independently coded transcripts from the staff and patient focus groups (*n* = 4). We developed consistent application of our codebook through iterative coding and consensus building. Next, four team members independently coded the remaining transcripts. We reached coding saturation when no new recommendations emerged and when recommendations were fully characterized.

During the coding process we refined the MTA framework for the current study with attention to features of residential treatment (e.g., conditions of the treatment environment that impact longer-term experiences; separation from family and community; opportunities related to treatment duration). We used a framework analysis to organize the data by recommendation type [37]. We sorted our data according to the refined MTAs and created sub-categories when constructs were multifaceted (e.g., increasing presence of Black staff and community members within the treatment setting). We coded and analyzed the transcripts in Dedoose.

## Results

The recommendations emergent from this work are categorized into five domains: [1] Enhance representation of Black people and people with lived experience in treatment settings; [2] Conduct staff training to effectively support Black patients in treatment; [3] Create accountability across organizational structures; [4] Increase awareness and responsiveness to trauma; and [5] Create a welcoming treatment environment, utilizing strength-based behavioral reinforcement and culturally tailored programming. We provide an exemplar quote for each recommendation and include a more comprehensive set of quotes in Table 1 to demonstrate the breadth of our findings.

### Recommendation one: Enhance representation of Black people and people with lived experience in treatment settings

**Table 1:** Quotes from focus groups and expert interviews informing recommendations

Residential Program Policy Recommendations	Exemplar Quotes
**1. Enhance representation of Black people and people with lived experience in treatment settings.**	
*1a. Hire staff with shared lived experiences to the patient population*	We were intentional about hiring more diverse staff… I wasn’t the only Black person at the table with my team. And that’s what we did which, oh my God, what a difference. And I remember our conversation like it feels like magic, doesn’t it? But it’s not. It’s intentional. Like this is what intention looks like. (Expert Interview 1) I started to feel comfortable because there was other African Americans here at the same time, whether it was a patient or staff. (Male, Patient FG 1) We have a client here who’s the only woman of color in her room. And there were certain things that she opened up to me about because she felt comfortable, because we can identify with one another… A lot of our patients have trauma already and they feel like in some areas of their life they’ve been discriminated [against]. So, to see somebody that they can identify with and relate with, I think it’s a great thing. (Female, Staff FG 1) But when you meet me, we’re both Blacks, so you’re gonna help me out, I shut down. I don’t like that. I feel like there has to be another way or you need me to help you out because you’re really having a hard time with your recovery. That’s different. It’s not that I’m gonna help you out because you’re Black. No. (Female, Staff FG 2)
*1b. Invite Black community members into the program*	It seems like you rarely see, even when commitments [AA meetings] come in, you would barely see African Americans come in. (Male, Patient FG 1) We sponsor a trip with our residents to Black Nativity. It’s amazing. The way when people come back – and how that was so moving to them. It also helps that our personal trainer that we use, who is an African American man. (Expert Interview 3)
2. **Conduct staff trainings to effectively support Black patients in treatment**.	
*2a. Train staff on the history of medical racism and its impact on clinical care for Black patients*	They think we’re aggressive, or they think we’re intimidating because the way we talk can be, I agree, can be to a Caucasian very intimidating but that’s not how we are. That’s not what we meant to do or say. Theres no middle ground…. They automatically have this vision of how we’re gonna take things or how we’re gonna respond to situations where our nature is aggressive and that just comes from our pride or whatever. And I don’t think that they are educated enough to know what’s the difference. (Male, Patient FG 2) I can’t help but notice that the White people often push the envelope. They do things that we’re asked not to do and while I’m being respectful and “obedient”, I see them breaking the rules all the time. (Female, Patient FG 1)
*2b. Use a racial equity/ cultural responsiveness lens to support patient/staff interaction*	[Race] plays a big role in terms of the experiences people come in with, the way people are treated or not treated, the way the barriers exist. I think also when racism or perceived racism is called out, the way staff respond and label people as difficult or challenging and don’t necessarily take the time to look within themselves or within what actually occurred. (Leadership FG) Our Black techs and nurses were able to engage people – like had a lower threshold for our Black patients and with our Black patients. Like, I don’t think once I heard a reason for a behavior plan or even a restraint and seclusion in one of our settings because a patient had an attitude or was acting up. It was because someone got physical, right? And there was a real threat to the environment. And even at that time never were they like, ‘You need to get out of here. You need to go.’ It was like, ‘No, we’re gonna manage this. We have protocols to manage this.’ And they did. (Expert Interview 1)
3. **Create accountability across organizational structures.**	
*3a. Develop protocols that reduce discretionary decision making to minimize potential bias*	Getting on the same page is essential and I think the best way to do that is to start from the top, which is the policy. I find it very strange that our policy says one thing and we’re doing something else. So, if we could sit in a setting like this with all staff, review and get to know our policies better, and figure out what works and what doesn’t work, I think that would be a good outcome for the patients that we serve. (Female, Staff FG 2) There’s a lot of work to be done, around rules, around subjective decision making. And, how do you make it there when it’s case by case, or, one person is getting it something and the other person isn’t, I guess [there’s] work to be done there… when a patient feels they’ve been discriminated against, the way our team responds. (Leadership FG) Anywhere you go, there’s going to be unwritten rules. They can give you a booklet just that, with rules and stuff like that. But you always got those rules that’s not written down. (Male, Patient FG 1) It was by accident. It wasn’t intentional. We had a situation where a patient lost a family member. He didn’t really specify. We were thinking it’s mom, ‘cause later on we found out otherwise. But he was allowed to go leave treatment, go to the funeral, and come back. And when he came back, he was bragging about it. Whereas, we had a patient of color who came in earlier than him and also experienced a significant loss. [The Black patient] was asking about going to the funeral for closure and he kinda got lost in translation. So, when he heard that from the other patient that was White, he lost it. (Female, Staff FG 2)
*3b. Create mechanisms to involve patients in refining program’s operations*	I think it would be helpful to hear and collect more information from our Black patients and let – to really determine what it is, where we’re missing the mark [on efforts to improve the program for Black patients]. (Expert Interview 1) The code of conduct that was developed by the community, by the members. So, if anything, we can always point to, well, those are the -- if something is happening in the center that shouldn’t be happening in the center, you just point to the code of conduct. (Expert Interview 5)
4. **Increase awareness and responsiveness to trauma.**	
*4a. Embed trauma-informed practices at every stage of treatment*	You have to remember that in a group program, in a group model, that everything that happens to each person happens in some way to everybody else too. (Expert Interview 2) I didn’t like the way they kicked out – they just discharged the Black girl that was in my room …She’s gone, and they might as well said, “Here, get your shit and go.” I was affected by how they got that girl out of there. (Female, Patient FG 1) When we started training with [public health academic] and being trauma informed, then it got better. And so, the model– we’re seeing more women are staying in treatment, because when I inherited the program, maybe 30% to 35% of the women were completing. (Expert Interview 2) [Two former treatment programs tailored for Black patients] provided, one could say, systemic racism 101, like how to recover from it. But because both places joyfully accepted White women, any woman, any person, a lot of White people got to hear from us, and we got to hear from them… [The director] understood that treatment had to engage women around racism as trauma…She understood that racism was something women were recovering from as well. And she also understood that the disease of addiction could lend itself to self-sabotage in that area. (Expert Interview 4)
5. **Create a welcoming treatment environment: utilize strength-based behavioral reinforcement and culturally tailored programming.**	
*5a. Support strong relationships between staff and patients*	I love the partnership that was developed between me and the treatment staff on how I would be participating in my treatment services. It’s like it was almost a contract. They asked me what I wanted to do and what did I want them to do in my efforts to reach my goals, one of staying off drugs and to try to do something in my life. So, they went beyond that and I think that that was so significant. (Expert Interview 6) *Discussion between two FG participants*: If you really want something from your counselor, you have to chase him down. You have to corner them. (Male, Participant 1) Coming in here, I was told that I had to meet with my counselor once a day, but I didn’t know I had to go looking for her. (Female, Participant 2) I had a counselor that I met with and I was here for three weeks and she finally came up to me and said, I’m your counselor too. I’m like, no, you’re not. I’ve been here three weeks. (Male, Participant 1) I’m leaving tomorrow. I still haven’t met one of my counselors that were assigned to me. Not once. (Female, Participant 2) (Patient FG 2)
*5b. Strengths-based treatment*	A lot of the staff here will approach you on a personal level and not on a clinical level because they know that everybody’s different and everybody has their struggle. And everybody has different personalities coming out of them at any given time. So, they approach you on your personal level. They touch your heart before they touch your mind. Because your mind will tell you that, oh, this one’s just picking on me, or I’m not doing nothing wrong or whatever. But they get you where you’re comfortable with them. (Male, Patient FG 2) Every day that a patient chooses to stay, that’s in a sense, success story for me… You can see the hope and everything in their eyes. If they willingly choose to go to further treatment just the whole demeanor and everything about them, like that’s growth, that’s success… Everyone’s story is different. So, what my work for ‘Jane’ might be a different success story for ‘Paul’, but it’s progress, progress not perfection. (Male, Staff FG 1) Having a culture that reflects who you are and where you come from in order to have a basis of understanding that you’re more than the role that you’ve been playing through the addiction process. And a lot of folks didn’t really understand that in the beginning until they started to see themselves come to life. (Expert Interview 2)
*5c. Culturally appropriate hair and skin care*	When you are a person of color, your hair texture is different. [Black patients] get very upset because we don’t have stuff for their hair and that’s their dignity. And then simple things like lotion and soap. (Female, Staff FG 2) The pushback that we get regarding the products for melanin is hospital-based, everything is standard… So when we are faced with these challenges of people of color saying, I need this certain shampoo and things like that, and then to be told to tell them well, it’s a hospital and we have like standard products. I just feel like that’s not ideal. It’s not fair. (Female, Staff FG 2) I love this place. I rate this place at least a nine because they do the most good in trying to meet your needs. But then again African Americans have a little bit more needs for health care, the products that they have. Healthcare is usually only products that Caucasians would use. I feel like the predominant program is geared to Caucasians, not geared to how are we gonna get the African Americans. (Male, Patient FG 2)
*5d. Offer evidence-based and holistic treatment options*	A big part of it becomes focused on humanity and dignity, and then focusing on good medical care, good quality and safety. Let’s make sure that the meds are following them from the [settings across the continuum of care]. So, good med management, and just really, really good care, making sure the groups are happening. (Expert Interview 3) I’ll explain the four medicines, allowing everybody to get that … And then my chief had given me some sweet grass … to pay respect to our ancestors, because our ancestors love the smell of it and to give it a cleanse of our spirit and allowing us to, stay grounded throughout the day and people really relax and chill on it. And then it’s nice too when it is that, because Indigenous people, Brown people, Black people … we never know exactly where it is that we come from, right. Unless it is the individual themselves telling us. So, being able to have exposure to that in a collective way. (Leadership FG) We would send them all to various church[es], the Catholic church, to Protestant church, the Baptist church, as a group, until they would decide if they wanted to go to church. If they didn’t want to go to church at all, that was fine too. But that was a part of the culture of the program. So, what you’re seeing is that on one side, we’re really trying to pull this disease away from them. And on the other side, we’re trying to build them up. So, the replacement is going in because of void, we have this big void here. (Expert Interview 2) We found that there was an opportunity to introduce things like music therapy, right? For certain populations, music can be really, really important part of that journey. And so, we did about a year ago partner with [local music school]. (Expert Interview 3) There was an arts room, it was just such an inclusive, beautiful, beautiful space – I mean, it was a rundown old motel we transitioned into a residential campus, right. But it was just that… because that was the autonomy, people had their own rooms… Patients could go in and out of – they would do all of our gardens. (Leadership FG) *Discussion between focus group participant and interviewer*: Yeah, it’s just the policies, the music policy as well. A lot of us have coping skills. I know for me personally, music helps me a lot, hearing pastor sermons helped me a lot. (Female, Participant) What is the policy on music and listening to certain – (Interviewer) It’s stripped of you…No access, none. (Female, Participant) (Patient FG 2)
*5e. Integrate family and community*	Family members would come for additional family counseling after the three visits…There’s so much recidivism in terms of family history around substance use disorder that is very prevalent in the Black community. So, that was another reason, is that what is the home going to look like when the family visits? Is there going to be alcohol there, even though that may not be their drug of choice? How are you going to be treating them? (Expert Interview 2) Family engagement and helping people reunite with their minor children, their adult children… [The treatment staff] would take risks, little risks at first that [social services] wouldn’t have approved of. It could be something like allowing the child to come to the center with the kinship foster grandmother, a real grandmother bringing the child to see the mother or father during Christmas holiday or something. (Expert Interview 4) Oftentimes [the women] would feel that they were abandoned by their family and they were holding on to that from part of the culture of their addiction and not really understanding that you took my children, you took all of these things from me. And they were very angry. And so, in order to help them to understand the dynamic around the importance of their family being involved. So, we would go through that for three weeks within that month to six weeks’ time. And eventually, the families would be able to come visit, and then they would be able to go home and visit. (Expert Interview 2) In the end, I would like to add that the restriction of visits and the use of electronic gadgets kind of helps them to be more focused on the recovery, right? Because this thing… can be really destructive, you know. So, they don’t get visit unless on exceptional cases. That is when visit is allowed. (Male, Staff FG 1)
*5 f. Prepare patients for life outside of treatment with a focus on community integration*	We had all sorts of people from the community come in who are peer supports and offer groups and one-on-ones. And it was beautiful work. We had apprenticeships for the patients who were interested. (Leadership FG) I’m going back to college. I’ve had the opportunity based on what this program has given me…They mapped this out for me so I can see myself becoming who I truly am inside and not just a drug addict or a homeless person on the streets running around. They’ve given me the opportunity to do so. And I’m taking it and I’m running with it. I’m going all the way. (Male, Patient FG 2) *On the benefit of patient’s having choices*: Working with people as they experience outcomes from their choices. If, when they have a negative experience, being that tool to support them to address and work through that negative experience, so that it ultimate, helping the patient to identify how to change that experience. So that it actually benefits them but not penalizing them for poor experiences. (Leadership FG) Many of them couldn’t read, and so we instituted a tutoring program that was connected to [local non-profit]. And so, we had a tutor that would come in twice a week and tutor them. (Expert Interview 2) I have heard about patients having some serious success. And one thing I do say about this specific facility, the placement rate is almost 100%. They place you. They get your extensions until you get placed. No, I haven’t seen not one person…leave this facility without going to a place, without having somewhere positive to go. (Male, Patient FG 2) My experience has been that when I do the footwork and I make the phone calls and I’m the one on the phone, the tablet, the computer, doing a lot of work and making a lot of calls, it’s a lot easier for me to get to my next step…It would be a lot better if they let us [access phones]. I understand that in some instances having a cell phone can be a distraction in a recovery setting. But maybe even if they gave it to us for a certain amount of time. (Male, Patient FG 2) Many times, folks don’t want to [stay in treatment]. They say, well, I’m good, you know. Okay, you’re good. So, what are you going to do when you leave? Are you going to go to any meetings? Are you going to go to patient counseling? …You got to keep trying to motivate people to go on to something that’s healthy and empowering them to maybe not want to keep up or go to jail or be in the streets or coming back to detoxes. (Expert Interview 6)

#### Hire staff with shared lived experiences to the patient population

*We have a client here who’s the only woman of color in her room. And there were certain things that she opened up to me about because she felt comfortable, because we can identify with one another… A lot of our patients have trauma already and they feel like in some areas of their life they’ve been discriminated [against]. So, to see somebody that they can identify with and relate with, I think it’s a great thing. (Female, staff FG 1).*

Black patient participants described discomfort when they were the only Black patient or attended a program with primarily White staff and expressed a sense of relief when this was not the case. Programs can change hiring practices to prioritize having a staff that reflects the racial composition of the patient population. This would entail hiring some Black staff across all positions (e.g., nurses, administrators). Racial concordance and lived SUD experience alone are not sufficient, though. Staff members’ perspectives, intentions, and behaviors must be attuned to the needs of Black patients. In addition, some mentioned that working with staff who had lived experience with SUDs increased their sense of comfort and treatment effectiveness.

#### Invite Black community members into the program

*It seems like you rarely see, even when commitments [AA meetings] come in, you would barely see African Americans come in. (Male, Patient FG 1)*

Residential treatment settings can invite Black people into treatment in informal ways. For example, many residential programs host 12-Step meetings, as described in the above quote. They can improve representation amongst guest speakers by developing relationships with 12-Step groups who have Black members. There are other opportunities to invite Black community members; for example, they can lead programming that supports holistic recovery goals (e.g., music, exercise, meditation).

### Recommendation two: Conduct staff trainings to effectively support Black patients in treatment

#### Train staff on the history of medical racism and its impact on clinical care for Black patients

*They think we’re aggressive, or they think we’re intimidating because the way we talk can be, I agree, can be to a Caucasian very intimidating. But that’s not how we are. That’s not what we meant to do or say. (Male, Patient FG 1)*

Black patients described being perceived as angry or threatening when they were in fact anxious or distressed. Participants agreed that staff responses to Black patients can be biased; they may mislabel a Black patient as “difficult,” rather than evaluate the situation holistically. Leadership expressed that staff were not always attuned to Black patients’ negative experiences related to authority in both medical and non-medical environments. Fostering greater knowledge of the history of anti-Black racism in society [38] and in healthcare is one way to increase staff’s ability to appropriately assess and respond to Black patients.

Toward this end, treatment settings can invest in staff development. Trainings and educational programming should be conducted at the time of hiring and on an ongoing basis. Trainings might cover topics such as: [1] the historical oppression of Black people in the U.S., including the War on Drugs and mass incarceration, and anti-Black racism; and [2] practical intervention skills, including interrupting microaggressions and reducing implicit bias.

#### Use a racial equity/ cultural responsiveness lens to support patient/staff interaction

*[Race] plays a big role in terms of the experiences people come in with, the way people are treated or not treated, the way the barriers exist. I think also when racism or perceived racism is called out, the way staff respond and label people as difficult or challenging and don’t necessarily take the time to look within themselves or within what actually occurred. (Leadership FG)*

Clinical supervision is necessary to support the range of patient - staff experiences. Introducing a focus on staff’s self-efficacy and skills when working with Black patients could reduce the incidence of negative experiences for Black patients. Treatment leadership must be committed to this goal, as it requires them to cultivate expertise in this area and promote reflection on race and racism by everyone who works in the program.

### Recommendation three: Create accountability across organizational structures

#### Develop protocols that reduce discretionary decision making to minimize potential bias

*Getting on the same page is essential and I think the best way to do that is to start from the top, which is the policy. I find it very strange that our policy says one thing and we’re doing something else. So, if we could sit in a setting like this with all staff, review and get to know our policies better, and figure out what works and what doesn’t work, I think that would be a good outcome for the patients that we serve. (Female, Staff FG 2)*

Residential treatment facilities are highly structured programs, yet staff frequently use discretion to decide whether a policy applies in a particular situation. While discretion is inevitably part of care delivery, discretionary decision-making can allow implicit bias to influence clinical judgement resulting in discriminatory outcomes.

Clear, detailed operational policies can improve equity in decision-making. Documenting scenarios where staff discretion is permitted and where it is not could also minimize bias. Leadership should make sure that staff apply policies – and discretion – consistently across all patients. To do this, leadership can familiarize staff with policies at onboarding, provide updates when policies change, and review practices and policies with staff regularly, as well as reviewing staff members’ clinical approach during clinical supervision.

#### Create mechanisms to involve patients in refining program’s operations

*I think it would be helpful to hear and collect more information from our Black patients and let – to really determine what it is, where we’re missing the mark [on efforts to improve the program for Black patients]. (Expert Interview 1)*

Programs can develop a process to gather and respond to patient feedback. Even though it is not possible to act on every suggestion, evaluating recommendations and demonstrating responsiveness are key so that patients know their voices are being heard. Patient-advisory groups, perhaps inclusive of both current and past patients, are one strategy to gather feedback and implement changes. Black patients should be well-represented in this group to help programs to address inequities in care.

### Recommendation four: Increase awareness and responsiveness to trauma

#### Embed trauma-informed practices at every stage of treatment

*[Two former treatment programs tailored for Black patients] provided, one could say, systemic racism 101, like how to recover from it. But because both places joyfully accepted White women, any woman, any person, a lot of White people got to hear from us, and we got to hear from them… [The director] understood that treatment had to engage women around racism as trauma…She understood that racism was something women were recovering from as well. And she also understood that the disease of addiction could lend itself to self-sabotage in that area. (Expert Interview 4)*

Increasing attention to trauma across the organization (e.g., screening for trauma, staff training) can facilitate examination of how it factors into patients’ addiction and recovery. In addition to traumas experienced among the general population, Black patients may also be impacted by racial trauma and discrimination. Treatment settings can provide psychoeducation on how racial trauma relates to addiction and recovery and can invite patients to discuss their experiences in individual counseling and groups.

### Recommendation five: Create a welcoming treatment environment: utilize strength-based behavioral reinforcement and culturally tailored programming

#### Support strong relationships between staff and patients

*Discussion about access to counseling: If you really want something from your counselor, you have to chase him down. You have to corner them. (Male, Participant 1)*

*Coming in here, I was told that I had to meet with my counselor once a day, but I didn’t know I had to go looking for her. (Female, Participant 2)*

*I had a counselor that I met with and I was here for three weeks and she finally came up to me and said, ‘I’m your counselor too.’ I’m like, no, you’re not. I’ve been here three weeks. (Male, Participant 1)*

*I’m leaving tomorrow. I still haven’t met one of my counselors that were assigned to me. Not once. (Female, Participant 2) (Patient FG 2)*.

Treatment settings demonstrate investment and foster success by connecting patients with staff and clinicians upon their arrival. The intake process should include an introduction to the counselor and instuctions on how to access the counselor. This connection provides patients with a clear point-person to answer questions, provide support, and address concerns.

#### *S*trengths-based treatment

*Every day that a patient chooses to stay, that’s in a sense, success story for me… You can see the hope and everything in their eyes. If they willingly choose to go to further treatment, just the whole demeanor and everything about them, like that’s growth, that’s success… Everyone’s story is different. So, what my work for ‘Jane’ might be a different success story for ‘Paul’, but it’s progress, progress not perfection. (Male, Staff FG 1)*

Treatment programs often rely on negative behavioral reinforcement, such as loss of privileges or threat of administrative discharge, to manage patient behavior or promote treatment engagement. Black patients explained that some aspects of treatment mimicked their experience of structural oppression in their daily lives, including previous carceral experiences. Integrating positive reinforcement, where successes are rewarded, is a needed transformation in treatment philosophy.

#### Culturally appropriate hair and skin care

*In reference to the short-term residential treatment program: Healthcare is usually only products that Caucasians would use. I feel like the predominant program is geared to Caucasians, not geared to how are we gonna get the African Americans. (Male, Patient FG 2)*

Residential treatment settings do not typically provide appropriate hair and skin care for Black patients. Supplying combs, soaps, and shampoos made for Black people communicates to Black patients that their needs are equally important to White patients’. Many participants expressed that self-care and feeling good in themselves was essential to their recovery. Without access to the necessary supplies, they were unable to fully embrace a positive recovery-oriented identity.

#### Offer evidence-based and holistic treatment options

*We found that there was an opportunity to introduce things like music therapy, right? For certain populations, music can be really, really important part of that journey. And so, we did about a year ago partner with [local music school]. (Expert Interview 3)*

Participants shared that they felt relatively less informed than White patients about their treatment options and that they were unable to make well-informed decisions about their care. To promote patient-centered care, treatment programs should provide psychoeducation about all evidence-based treatments. In addition to evidence-based interventions, residential programs can incorporate holistic modalities. For example, participants shared that access to music was important to their recovery journey.

In addition to formal offerings, participants shared that having physical space for spiritual and religious practices would improve their treatment experience. They explained that a holistic approach – where individuals’ spiritual wholeness was considered – more aptly addressed recovery in a cultural context accessible to many Black people.

#### Integrate family and community

*Family members would come for additional family counseling after the three visits… There’s so much recidivism in terms of family history around substance use disorder that is very prevalent in the Black community. So, that was another reason – is that, what is the home going to look like when the family visits? Is there going to be alcohol there, even though that may not be their drug of choice? How are you going to be treating them? (Expert Interview 2)*

Addiction and recovery occur in the context of community, but treatment settings often conceptualize treatment at the individual patient level. Treatment facilities can create opportunities for family members to participate in treatment, including family counseling, psychoeducation for family members, and the celebration of treatment milestones. Incorporating nuclear and extended family may make treatment more relevant for Black patients and lay the foundation for success after their discharge.

#### Prepare patients for life outside of treatment with a focus on community integration

*I’m going back to college. I’ve had the opportunity based on what this program has given me…They mapped this out for me so I can see myself becoming who I truly am inside and not just a drug addict or a homeless person on the streets running around. They’ve given me the opportunity to do so. And I’m taking it and I’m running with it. I’m going all the way. (Male, Patient FG 2)*

Patients in residential treatment need to be connected with the necessary resources to re-establish themselves in the community. Pragmatic assistance might include retrieval of important documentation (e.g., birth certificate) or reinstating licenses. During discharge planning, treatment staff should arrange follow up care in patients’ communities. And finally, many participants expressed need for economic stability. While treatment programs may not be able to address this directly, they can build relationships with social services and community resources that offer educational and employment opportunities.

## Discussion

Our recommendations for residential treatment programs are informed by interviews with experts in the addiction treatment field and focus groups with leadership, frontline staff, and patients in a short-term residential treatment program. Accountability and equity are at the core of these recommendations - accountability to equity in hiring, in the equitable use of discretion in clinical decision making, and the provision of treatment modalities, among others.

### Staff hiring and training

Participants in our study reported that racial concordance between patients and their care team often enhanced patient comfort and safety, a preference well documented in other addiction research [39, 40]. One of the key experts (Table 1) shared that when her program was staffed by Black individuals, she noticed they were able to effectively deescalate and support Black patients during moments of distress, rather than turn to behavioral evaluation or restraint. She also explained how impactful Black leadership can be. She described it as “magic” and evidence of what can be possible with intentionality. These examples illustrate potential benefits of hiring Black individuals in leadership and staff positions [21]. 

Participants in our study shared that staff treated them differently and enforced policies differently due to their race. The use of discretion is understandable and necessary. However, implicit bias and racial discrimination may drive inequitable decisions when staff are uninformed about the manifestations of racism in addiction treatment or when they are ill equipped to navigate it [22, 39–41]. Educating leadership and staff about racism and its impact on Black patients (e.g., treatment drop out) [30] is an important first step to improve their ability to provide compassionate and effective care [42, 43]. Future work is needed to test which trainings change treatment experiences and outcomes for Black patients.

### Trauma informed care

Previous research has described the relationships between structural racism, racial trauma and Black patients’ treatment seeking and experiences [42, 44]. Participants spoke about the often-overlooked role of trauma and racial trauma in the development and treatment of addiction. Key experts who ran treatment programs tailored for Black patients (both now closed) shared how they addressed trauma and racism. They reflected on the success of these efforts in improving patients’ self-esteem, patients’ commitment to recovery, and the community’s wellbeing. When Black people enter treatment, providers should be prepared to support all areas of their recovery, including the roles of racism and trauma [40]. New measures to assess racial trauma are available and can guide clinical intervention [45, 46]. In addition to racial trauma, programs should regularly screen and provide appropriate treatment or referrals for co-occurring trauma and PTSD [47, 48], as trauma-related symptoms affect addiction treatment outcomes and retention [49]. 

### Culturally tailored, strengths-based treatment

Providing a culturally appropriate environment and treatment offerings can make treatment more appealing, effective, and equitable for Black patients. Our findings point toward the importance of including multiple modalities including music therapy, theater, Indigenous practices (e.g., connection to elders), and spirituality or organized religion [50–55]. Modifying the environment to better support Black patients does not diminish the relevance of the space for other clients. As one leader of a former program stated, her program served a multiracial patient population and everyone benefitted from the holistic approach to recovery.

A strengths-based approach to treatment is especially important for Black patients so that the treatment environment does not replicate the oppression of structural racism [21, 42]. Treatment providers and patients in our study described how valuable it is to approach patients with open-mindedness and “the benefit of the doubt.” Programs too often rely on negative reinforcement, which can lead to administrative discharge and overlook opportunities for patient growth.

In addition to policies and attitudes, our team recommends the integration of evidence-based strengths-based modalities. For example, contingency management (CM) is a highly acceptable and effective modality [56] that may particularly benefit Black patients when it replaces punishment-based behavioral modification (i.e., negative reinforcement). CM is associated with improved self-efficacy and psychological wellbeing and improved patients’ relationships with treatment providers and family members [57, 58]. Thus, the benefits of CM may extend to the psychosocial features of recovery, as well as those related to basic needs [57]. 

### Limitations

This qualitative study was conducted in a single city, and our study population included affiliates of a short-term residential treatment facility and local experts on addiction treatment for Black patients. Our findings are transferable to residential treatment settings in similar geographic and cultural contexts. Further research is likely necessary to adapt our recommendations for other treatment settings or regions. While our recommendations are designed to be broadly applicable to Black patients, the Black patient population is heterogenous. Multiple intersecting identities influence treatment experiences and recovery needs for Black patients [59]. Each setting should be mindful of how their policies and practices support their patients’ intersecting identities.

## Conclusion

Leadership and staff face the difficult but important responsibility to implement these findings in a way that does not create divisions among patients or staff. We encourage treatment programs to value and prioritize changes that benefit underserved patient populations while protecting their programming [58]. The precarity of the political and social environment make it especially important that treatment programs make adaptations to support Black patients while continuing to support the needs of other patient populations.

## Supplementary Information

Below is the link to the electronic supplementary material.

Supplementary Material 1

## Data Availability

The authors will provide a summary of the data upon reasonable request.
