# Supplementary material for: Policy and practice recommendations for residential addiction treatment: a qualitative investigation on increasing equity in treatment for Black patients
Source: Addict Sci Clin Pract. 2026 Jul 28;21:56. doi: 10.1186/s13722-026-00677-z (PMC13412139; doi:10.1186/s13722-026-00677-z)
Supplement: Supplementary file 1 — Supplementary Material 1 [file 13722_2026_677_MOESM1_ESM.docx]

| Preamble for focus groups and interviews: |
| --- |
| Introduction:  Thank you for agreeing to speak with us today. We are exploring ways to improve addiction treatment specifically in addiction residential programs.  We are particularly interested in understanding how programs’ operational policies can promote or prevent high quality care for Black patients. We want to learn more about your experiences and organizational policies at this program **(or at your program or a past program)** to develop model operational policies that current and future programs can adopt. By operational policies we mean; rules and guidelines that define how your program operates on a daily basis. They help achieve consistency, quality, efficiency, compliance and customer satisfaction. Would you like an example of an operational policy?  A few examples are:   - Family members are only allowed to visit clients during a monthly-supervised group meeting. - Patient allegations of mistreatment by staff are managed case by case with consequences. |

| CSS Patient Focus Group Guide | Individual Patient Interview Guide |  |
| --- | --- | --- |
| 1. Let’s start out with how you got to this program. For example, how’d you hear about this program? How was your admission to this program? 2. Tell me about your experience so far in the addiction treatment (CSS) program here:    1. How would you rate your experience on a scale from 1-10 with 10 being the highest?    2. What does success mean to you in this current program you’re in? 3. Can you share any examples of patient success you’ve seen in this program or after being in the program? 4. Have you heard of patient success stories from this program? Would you be able to share in what way they have been successful and what aspects of the program contributed to their success? 5. What about examples when patients had less success in this program or after being in the program? 6. How often do patients successfully complete the program vs have an unplanned discharge? 7. How does this differ for Black patients versus other patients? 8. Okay, now lets talk about how they run the program here. What are the policies, guidelines or rules that you think help patients succeed in the program? Which policies do you think make it harder for them to succeed? 9. Which of these guidelines are written versus unspoken? 10. How flexible are the staff with upholding expectations of these policies? 11. When have you seen staff be more strict? Less strict? 12. From your perspective, have you seen or heard directly about staff being more strict with Black patients compared to other patients who are not Black? 13. How would you change the policies/guidelines? | 1. Can you tell me about your experience at XXX addiction rehabilitation program 2. Was there anything special or unusual about this program? 3. Was there a specific group you thought this program was tailored to? 4. What did you like most about this program? 5. What did you like least about this program? 6. Did you share these things with the program? If so, do you know if the program made any changes in response to feedback? 7. Did the addiction treatment experience at XXX feel like it was tailored to your needs? If yes, how?  If no, why not? 8. What differences did you see in the treatment that was offered based on race? 9. Have you noticed major differences in your experiences at other treatment programs, with regard to how comfortable it felt as a Black person to receive treatment there? If so, what were they? 10. In what way do you think Black identity has had an impact on your experience receiving addiction treatment? 11. During your intake or referral process, did anyone describe what the experience at this program might be like? If so, how was that helpful? 12. What suggestions would you make to create a more positive and effective experience in addiction treatment? |  |
| Staff & Leadership Focus Group Script | Program Administrator Interview Script | |
| 1. Where do patients get referred from? Self or other referrals? Geographics? 2. What is the intake process? 3. What are your expectations for patients while they are in the program? How do they differ by race? 4. What does patient success during the program look like to you? 5. What operational policies do you think best support patient success? 6. Which operational policies are the most challenging to implement? 7. Which policies that you see are being enforced differently for Black patients compared to other patients? 8. What differences do you see in how Black patients are being treated compared to other patients? 9. How does the staff and patient being of the same race play a role in this experience? 10. What opportunities do patients have for expressing feedback regarding their experience in the CSS program? 11. How do you define success for patients after they leave the program? | 1. Can you tell us about your program? What types of addiction treatment services does your program offer? 2. Is there a specific population that your program is tailored to? How would you estimate the breakdown of your patients by gender and race? 3. Is your program tailored in any particular way to meet the needs of Black patients? If so, how?  (If not, skip) 4. How have those methods of tailoring your program affected Black patients? Or other patients? 5. Are these practices or policies? 6. How do you see your approach as different from other addiction residential programs? 7. Can you share an example of a time where the implementation of a one of your operational policies related to tailoring your program toward Black patients was successful or challenging? 8. If there was success, ask why they thought it was successful (or challenging). 9. What policies contributed towards that person’s success (or challenge)? 10. Do you do surveys or provide other feedback mechanisms for clients? 11. Can you tell us about those—when and how do patients give feedback? 12. What happens in response to this feedback? 13. Do patients know about what happens in response to their feedback? How? 14. What operational policies do you see as having improved the treatment or experience of Black patients? How/why? What policies had a negative effect on Black patients? How/why? 15. What policies you feel supported treatment for black patients?   We reached out to you because XXX program has been highlighted as one that may have had policies that were particularly impactful on addiction recovery for Black patients.   1. Since you originally launched the program, what changes have you made in order to improve the experiences of Black patients? 2. What other efforts could have been made to improve the experiences of Black patients? 3. What were the most common suggestions you heard from Black patients? 4. How do you see the experience of Black patients in your facility differing from the experience of White patients? | |
